# Breast cancer screening and early diagnosis in China: a systematic review and meta-analysis on 10.72 million women

**DOI:** 10.1186/s12905-024-02924-4

**Published:** 2024-02-07

**Authors:** Mengdan Li, Hongying Wang, Ning Qu, Haozhe Piao, Bo Zhu

**Affiliations:** 1 Department of Liaoning Office for Cancer Prevention and Control, Cancer Hospital of China Medical University, Liaoning Cancer Hospital & Institute, No.44 Xiaoheyan Road, Dadong District, Shenyang, Liaoning 110042 China; 2 Department of School of Public Health, China Medical University, Shenyang, Liaoning 110122 China; 3 Department of Radiology, Cancer Hospital of China Medical University, Liaoning Cancer Hospital & Institute, Shenyang, Liaoning 110042 China; 4 Department of Neurosurgery, Cancer Hospital of China Medical University, Liaoning Cancer Hospital & Institute, No.44 Xiaoheyan Road, Dadong District, Shenyang, Liaoning 110042 China

**Keywords:** Breast cancer, Opportunistic screening, Population screening, Early diagnosis

## Abstract

**Background:**

The incidence of breast cancer among Chinese women has gradually increased in recent years. This study aims to analyze the situation of breast cancer screening programs in China and compare the cancer detection rates (CDRs), early-stage cancer detection rates (ECDRs), and the proportions of early-stage cancer among different programs.

**Methods:**

We conducted a systematic review and meta-analysis of studies in multiple literature databases. Studies that were published between January 1, 2010 and June 30, 2023 were retrieved. A random effects model was employed to pool the single group rate, and subgroup analyses were carried out based on screening model, time, process, age, population, and follow-up method.

**Results:**

A total of 35 studies, including 47 databases, satisfied the inclusion criteria. Compared with opportunistic screening, the CDR (1.32‰, 95% CI: 1.10‰–1.56‰) and the ECDR (0.82‰, 95% CI: 0.66‰–0.99‰) were lower for population screening, but the proportion of early-stage breast cancer (80.17%, 95% CI: 71.40%–87.83%) was higher. In subgroup analysis, the CDR of population screening was higher in the urban group (2.28‰, 95% CI: 1.70‰–2.94‰), in the breast ultrasonography (BUS) in parallel with mammography (MAM) group (3.29‰, 95% CI: 2.48‰–4.21‰), and in the second screening follow-up group (2.47‰, 95% CI: 1.64‰–3.47‰), and the proportion of early-stage breast cancer was 85.70% (95% CI: 68.73%–97.29%), 88.18% (95% CI: 84.53%–91.46%), and 90.05% (95% CI: 84.07%–94.95%), respectively.

**Conclusion:**

There were significant differences between opportunistic and population screening programs. The results of these population screening studies were influenced by the screening process, age, population, and follow-up method. In the future, China should carry out more high-quality and systematic population-based screening programs to improve screening coverage and service.

**Supplementary Information:**

The online version contains supplementary material available at 10.1186/s12905-024-02924-4.

## Background

Breast cancer is the most common malignant tumor in the world [1]. In China, the incidence of breast cancer and the disease burden continue to increase [2]. Improving the early diagnosis of breast cancer followed by effective treatment is an effective measure to reduce breast cancer mortality [3–5]. Western countries began to standardize the breast cancer screening process earlier than China, and have successively implemented screening programs [6–8]. For large-scale cancer screening, cases must be effectively detected, especially early cases [9].

In the past 10 years, the provinces and cities in China have also launched several population-based breast cancer screening programs successively. Notably, two national cancer screening programs [10, 11] have persisted and yielded considerable social benefits. However, due to the large, widely dispersed population and shortage of equipment in China, it is difficult to unify breast cancer screening strategies in different programs. Meanwhile, some problems were exposed. For example, the starting age was not standardized, some screening programs had a short duration and no follow-up surveys, and the types of screening equipment were different in some institutions.

Published studies on breast cancer screening in China mainly focused on risk factors and screening techniques. Most of the data were from a single province, part of a region, or a single program. There is no comprehensive analysis of all studies, let alone analysis of early diagnosis. We aimed to analyze the current situation of breast cancer screening in China. Therefore, in the present study, we systematically analyzed the population and opportunistic breast cancer screening programs in China, and compared the cancer detection rates (CDRs), early-stage cancer detection rates (ECDRs), and the proportions of early-stage cancer. Subgroup analysis of population screening was conducted based on screening time, screening process, target population, and follow-up method.

## Methods

The review protocol was registered in the Open Science Framework (10.17605/OSF.IO/EABPH).

### Search strategy

We searched relevant articles in databases including PubMed, EMBASE, Web of Science, China National Knowledge Infrastructure (CNKI), Chinese Scientific Journals Full Text Database (CQVIP), and Wanfang Data. Articles published between January 1, 2010 and June 30, 2023 were considered for inclusion. The search keywords included “breast cancer” OR “breast tumors” AND “screening” AND “China” OR “Chinese” (Table S1). In addition, we manually searched systematic reviews and references. This study was conducted and reported in accordance with the Preferred Reporting Items for Systematic Reviews and Meta-Analyses (PRISMA) statement [12] (Table S2, PRISMA checklist).

### Study selection

A literature database was created to retrieve relevant studies and exclude duplicate studies by Endnote® (version X6; Thomson Reuters, Inc., Philadelphia, PA) bibliographic software. In order to prevent bias, two authors (LMD and ZB) independently screened the titles and abstracts. Any disagreements were resolved by discussion with the third author. Finally, the preliminary selected articles were examined in full texts, and irrelevant articles were excluded according to the inclusion and exclusion criteria.

The inclusion and exclusion criteria were as follows: (1) the subjects were from mainland China and voluntarily participated in breast cancer screening; (2) studies of patients with breast cancer or precancerous lesions were excluded; (3) the overall sample size was ≥ 1000; (4) the screening process, methods, and detection indicators were clearly defined, especially with respect to the detection rates of breast cancer and early-stage breast cancer; and (5) when two or more studies were conducted in the same study population, the most recent article or the article with the largest sample size was included.

### Quality assessment

To assessed the quality and validity of the included studies, a modified quality assessment tool based on ten aspects was used [13]. For each aspect, a score of 0 (high risk) or 1 (low risk) was given, so the total score ranged from 0 to 10. Studies with 8 to 10 points were considered to be of high quality, studies with 4 to 7 points were considered to be of moderate quality. Furthermore, studies with points below 7 were considered low quality and excluded from the research.

### Data extraction

The included studies were read in detail by two authors (LMD and ZB). Moreover, the following variables were extracted: first author, year of publication, characteristics of the screening programs (screening mode, screening time, target population, province, age range, screening process, follow-up method), the number of screening participants, the number of detected breast cancers and early breast cancers, etc.

### Statistical analysis

Stata (Version 14.0; Stata Corp., College Station, TX) was used for the pooled analysis. The random effects model was used to combine the results (CDRs, ECDRs, and the proportions of early-stage cancer) and 95% CI. The heterogeneity of the selected studies was assessed using the *I*^*2*^ index.

Population screening refers to the systematic and organized examination conducted on all women in the target group, whether at a national, region, or unit level. Opportunistic screening involves women voluntarily choosing to undergo examination at medical institutions or as recommendation during routine medical consultations**.** Furthermore, subgroup analyses were carried out to explore the main heterogeneity of population screening by screening time (< 2012 and ≥ 2012 year), screening age (< 40, 40–49, 50–59 and ≥ 60 years old), residence (urban/rural), geographical region (north and south), Human development index (HDI) (< 0.75, 0.79–0.75 and ≥ 0.8), screening process, and follow-up method. According to the population screening methods, the screening process could be divided into three main categories: (i) subjects underwent clinical breast examination (CBE) as initial screening, some of them followed by breast ultrasonography (BUS) or mammography (MAM) according to the results; (ii) subjects underwent BUS as initial screening, some of them followed by MAM according to the results; and (iii) subjects underwent BUS in parallel with MAM as initial screening. Follow-up involves tracking women who received positive screening results through various methods to obtain the final diagnosis and results. We divided the studies into three types: (i) no follow-up; (ii) inquiry follow-up by telephone or interview after 1 year, and (iii) second screening after 1 year.

## Results

### Literature search and study characteristics

**Fig. 1:**
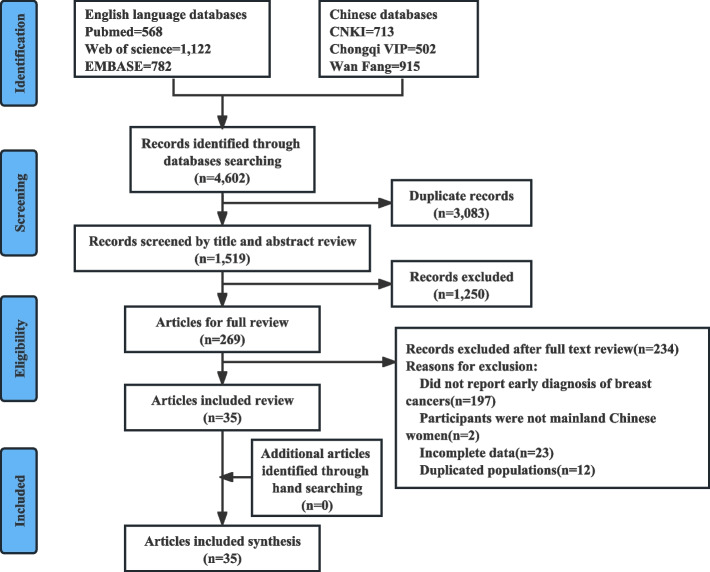
Flowchart of the screening process in our meta-analysis

According to the process, a total of 4,602 studies were initial found in the databases. During the screening stage, 3,083 studies were excluded due to duplication, while an additional 1,250 studies were excluded based on title and abstract reviews. In the eligibility evaluation stage, 269 studies were accessed in full text, and 234 studies were excluded by considering the inclusion and exclusion criteria. Finally, 35 studies [14–48] (five in English and 30 in Chinese), 47 databases, and a total of 12,984,958 participants were included in the analysis. A flowchart of the screening process is shown in Fig. 1.

Of these 47 databases, 39 were from population screening and 8 were from opportunistic screening. The databases covered a number of provinces in China, of which three were national screening programs and five were multi-center screening programs. We conducted subgroup analysis of the population screening databases. The screening population of 19 databases were urban population and 20 were rural population. The screening times of 21 databases were before 2012 and 18 were in or after 2012. Based on the screening process of the population screening databases. 22 were from BUS followed by MAM, and 12 were from BUS in parallel with MAM as initial screening. Other characteristics of the databases are summarized in Table 1.

**Table 1:** Characteristics of the included studies (China, 2010–2023)

Author	Publication time	Screening model	Screening time	Participants	Residence	Province	Screening age	Screening process	The quality score
Yang, S [14]	2023	Opportunistic	2020.1–2021.12	3,874	Urban	Heilongjiang	20–70	BUS followed by MAM	7
Wu, SQ [15]	2023	Population	2013–2021	23,009	Urban	Hebei	40–74	BUS in parallel with MAM	9
Wu, L [16]	2023	Population	2021	2,231,092	Urban/ Rural	Guangdong	35–64	BUS followed by MAM	8
Han, T [17]	2023	Population	2021.8–2022.3	5,974	Rural	Shanxi	35–64	BUS followed by MAM	8
Wu, JM [18]	2022	Population	2018	1,156,287	Rural	Hebei, Henan, Hubei, Guangxi, Xinjiang, Gansu	35–64	BUS followed by MAM	9
Zhou, TH [19]	2021	Population	2014–2018	11,752	Urban	Xinjiang	40–74	BUS in parallel with MAM	8
Xiao, BL [20]	2021	Population	2017.1–2019.12	143,383	Rural	Guangdong	26–68	BUS followed by MAM	9
Shen, SJ [21]	2021	Opportunistic/ Population	2014.1–2016.12	20,080	Urban	Hebei and other 10 provinces	35–75	CBE followed by BUS or MAM /BUS followed by MAM	7
Shang, GXH [22]	2021	Population	2017–2020	121,916	Rural	Fujian	35–64	BUS followed by MAM	9
Ma, L [23]	2021	Population	2015	1,501,753	Rural	National	35–64	BUS followed by MAM	9
Lin, HZ [24]	2021	Opportunistic/ Population	2014	48,688	Rural	Guangdong	35–64	CBE followed by BUS or MAM /BUS followed by MAM	8
Zhao, YX [25]	2020	Population	2014	1,373,524	Rural	National	35–64	BUS followed by MAM	9
Yang, YP [26]	2020	Population	2013–2018	28,621	Urban	Guangdong	22–89	BUS in parallel with MAM	8
Yang, L [27]	2020	Population	2014–2019	8,353	Urban	Beijing	40–69	BUS in parallel with MAM	8
Wang, R [28]	2020	Population	2018.12–2019.5	27,406	Urban	Xinjiang	35–64	BUS followed by MAM	8
Liu, GM [29]	2020	Population	2013–2019	144,151	Rural	Beijing	35–64	BUS followed by MAM	9
Xiong, LL [30]	2020	Population	2016–2018	3,151,679	Rural	Hunan	35–64	BUS followed by MAM	9
Huang, XX [31]	2020	Population	2015–2018	438,893	Rural	Fujian	35–64	BUS followed by MAM	9
Ding, ST [32]	2020	Population	2013.6–2016.12	24,693	Urban	Beijing	35–64	BUS followed by MAM	8
Wu, MQ [33]	2018	Population	2014.12–2015.5	5,066	Urban	Xinjiang	30–60	BUS followed by MAM	8
Shen, SY [34]	2017	Opportunistic/ Population	2015.1–12	2,062	Urban	Guangdong	35–88	BUS in parallel with MAM	7
Huang, YB [35]	2016	Population	2008.3–2011.12	1,226,714	Urban/ Rural	National	35–59	CBE followed by BUS or MAM	9
Shen, S [36]	2015	Population	2008.11–2010.11	12,519	Rural	Hebei and other 7 provinces	30–65	MAM/ BUS followed by MAM/ BUS in parallel with MAM	8
Mo, M [37]	2015	Opportunistic	2008.5–2010.10	104,809	Urban	Shanghai	35–74	BUS/MAM/BUS followed by MAM	9
Ma, HM [38]	2015	Population	2008–2011	64,864	Urban/ Rural	Shandong	35–64	BUS followed by MAM/ BUS in parallel with MAM	8
Yu, HY [39]	2013	Population	2006–2011	10,767	Urban	Guangdong	30–78	BUS followed by MAM	8
Xu, J [40]	2013	Population	2010.1–2011.12	284,168	Rural	Guangdong	35–59	BUS followed by MAM	9
Shi, SD [41]	2013	Population	2009–2011	6,122	Rural	Shanxi	30–59	CBE followed by BUS or MAM	8
Mo, M [42]	2013	Population	2008.5–2012.9	14,464	Urban	Shanghai	35–74	BUS in parallel with MAM	9
Gong, YH [43]	2013	Population	2008–2011.12	70,292	Urban	Jiangsu	18–92	CBE followed by BUS or MAM	9
Yang, ZH [44]	2012	Population	2008.7–2009.9	22,960	Urban	Liaoning, Tianjin, Jiangxi Shandong	45–65	BUS in parallel with MAM	8
Kuang, XM [45]	2012	Opportunistic	2011	5,722	Urban	Guangdong	17–69	CBE followed by BUS or MAM	7
Huang, Y [46]	2012	Opportunistic/ Population	2009.3–2011.7	3,028	Urban	Chengdu	25-	BUS in parallel with MAM	7
Han, LL [47]	2011	Population	2008–2009	568,000	Rural	Beijing	40–60	BUS followed by MAM	9
Xu, GW [48]	2010	Opportunistic/ Population	2005–2006	118,273	Urban	Hebei and other 15 provinces	35–70	BUS in parallel with MAM	7

*Abbreviations:* *CBE* clinical breast examination, *BUS* breast ultrasonography, *MAM* mammography

### Risk of bias

We assessed the quality of the studies using the modified quality assessment tool. The scores ranged from 7 to 10. Of the 35 studies, six studies were considered as being of medium quality and 29 studies were considered as being of high quality. After our evaluation, each included study established its own quality control program, and required all physicians and technicians to be trained accordingly (the physicians were responsible for making the diagnosis). Details are shown in Table 1.

### Comparison of breast cancer screening effect between opportunistic and population screening programs in China

The detailed data on breast cancer detection by screening model are summarized in Table 2. Overall, the CDR in the opportunistic screening group with a sample size of 224,240 was 11.99‰ (95% CI: 5.14‰–21.57‰; *I*^2^ = 98%), and in the population screening group with a sample size of 12,760,718 was 1.32‰ (95% CI: 1.10‰–1.56‰; *I*^2^ = 99%). When we defined TNM stage 0–II as early-stage breast cancer, the ECDR of opportunistic screening group was 4.90‰ (95% CI: 1.02‰–11.37‰) and the proportion of early-stage cancer was 72.42% (95% CI: 57.28%–85.57%); the ECDR of populations screening group was 0.82‰ (95% CI: 0.66‰–0.99‰) and the proportion of early-stage cancer was 80.17% (95% CI: 71.40%–87.83%). The forest plots of pooled data by screening model are shown in Figure S1-3.

**Table 2:** The pooled results of breast cancer screening programs by screening model (China, 2010–2023)

Screening model	Databases	Total	Events	Estimates (95% CI)
CDR				
Opportunistic	8	224,240	1,395	11.99‰ (5.14‰–21.57‰)
Population	39	12,760,718	10,408	1.32‰ (1.10‰–1.56‰)
ECDR				
Opportunistic	6	115,557	660	4.90‰ (1.02‰–11.37‰)
Population	33	12,662,808	5,918	0.82‰ (0.66‰–0.99‰)
The proportion of early-stage cancer				
Opportunistic	6	806	660	72.42% (57.28%–85.57%)
Population	33	9,583	5,918	80.17% (71.40%–87.83%)

*Abbreviations:* *CDR* cancer detection rate, it was calculated as the number of detected breast cancers divided by the number of participants, *ECDR* Early-stage cancer detection rate, it was calculated as the number of early-stage (0–II) breast cancers divided by the total number of participants; The proportion of early-stage cancer, it was calculated as the number of early-stage (0–II) breast cancers divided by the total number of TNM stage breast cancers

### The breast cancer screening effect based on population in population screening programs

We further divided the population screening programs into urban women and rural women. The CDR of urban women (2.28‰, 95% CI: 1.70‰–2.94‰) was higher than that of rural women (0.70‰, 95% CI: 0.57‰–0.3‰) (Table 3). At the same time, more stage 0–II breast cancer was detected by population screening in urban women, with the ECDR of 1.60‰ (95% CI: 1.19‰–2.06‰) and the proportion of early-stage cancer of 85.70% (95% CI: 68.73%–97.29%) (Table 4). Regarding age, with the increasing of screening age, the CDR gradually increased, and in the ≥ 60 age groups, the CDR increased to 1.76‰ (95% CI: 1.03‰–2.68‰) (Table 3). To explore variations in breast cancer screening programs across different populations, we conducted subgroup analyses based on geographic location and the provinces’ HDI sizes. The results showed a slightly higher CDR in north China compared to south China, although the difference was not obvious (Table S3). Additionally, within urban population, regions with an HDI ≥ 0.8 exhibited relatively higher CDR and ECDR (Table S3-5).

**Table 3:** The pooled breast cancer detection rates in different subgroups of population screening programs (China, 2010–2023)

Subgroup		Databases	Total	Events	CDRs, ‰ (95% CI)
Residence	Urban	19	2,594,325	4,348	2.28 (1.70–2.94)
	Rural	20	10,164,379	6,060	0.70 (0.57–0.83)
Screening age	< 40	11	682,816	218	0.24 (0.13–0.40)
	40–49	11	1,385,665	1,081	1.11 (0.85–1.39)
	50–59	11	947,955	862	1.36 (1.06–1.70)
	≥ 60	9	288,840	258	1.76 (1.03–2.68)
Screening process	CBE followed by BUS or MAM	4	1,303,128	679	0.48 (0.40–0.56)
	BUS followed by MAM	22	11,278,524	9,223	0.94 (0.70–1.20)
	BUS in parallel with MAM	12	172,882	501	3.29 (2.48–4.21)
Screening time	Before2012	18	2,325,418	1,468	1.38 (1.08–1.71)
	In and after2012	21	10,433,286	8,940	1.26 (0.96–1.60)
Follow-up method	No	16	8,049,956	6485	0.81 (0.51–1.17)
	Interview	13	4,633,43	3,726	1.41 (1.15–1.69)
	Second screening	10	75,311	197	2.54 (1.65–3.61)

*Abbreviations:* *CDRs* cancer detection rates, it was calculated as the number of detected breast cancers divided by the number of participants

**Table 4:** The pooled early-stage (0–II) breast cancer detection in different subgroups of population screening programs (China, 2010–2023)

Subgroup	Databases	ECDRs, ‰			The proportion of early-stage breast cancer, %		
		Total	Events	Estimates (95% CI)	Total	Events	Estimates (95%CI)
Residence							
Urban	15	2,546,483	1,863	1.60 (1.19–2.06)	4,141	1,863	85.70 (68.73–97.29)
Rural	18	10,114,311	4,055	0.44 (0.31–0.60)	5,442	4,055	75.08 (66.16–83.15)
Screening process							
CBE followed by BUS or MAM	4	1,303,128	438	0.32 (0.26–0.38)	514	438	89.30 (85.79–92.48)
BUS followed by MAM	18	11,191,995	5,116	0.52 (0.37–0.69)	8,648	5,116	74.18 (61.87–84.92)
BUS in parallel with MAM	10	161,501	360	2.49 (1.89–3.16)	416	360	88.18 (84.53–91.46)
Study period							
Before2012	14	2,260,554	887	1.12 (0.79–1.50)	1,181	887	82.94 (69.47–93.52)
In and after2012	19	10,400,240	5,031	0.69 (0.52–0.89)	8,402	5,031	78.18 (66.41–88.19)
Follow-up method							
No	15	8,012,762	2,907	0.46 (0.30–0.64)	6,041	2,907	71.10 (59.07–81.95)
Interview	11	4,600,391	2,874	0.85 (0.65–1.06)	3,387	2,874	85.91 (84.65–87.14)
Second screening	7	47,641	137	2.62 (1.66–3.78)	155	137	90.05 (84.07–94.95)

*Abbreviations:* *ECDR* Early-stage cancer detection rate, it was calculated as the number of early-stage (0–II) breast cancers divided by the total number of participants; The proportion of early-stage cancer, it was calculated as the number of early-stage (0–II) breast cancers divided by the total number of TNM stage breast cancers

### The breast cancer screening effect based on screening process in population screening programs

The potential sources of population screening heterogeneity were assessed by estimating the detection rates based on different screening process. Overall, the CDR was 3.29‰ (95% CI: 2.48‰–4.21‰) in the BUS in parallel with MAM screening group, which was higher than in the CBE followed by BUS or MAM group (0.48‰, 95% CI: 0.40‰–0.56‰) and in the BUS followed by MAM group (0.94‰, 95% CI: 0.70‰–1.20‰) (Table 3); in the early detection of breast cancer, the BUS in parallel with MAM screening group also had a significant advantage. The ECDR was 2.49‰ (95% CI: 1.89‰–3.16‰) (Table 4).

### The breast cancer screening effect based on screening time in population screening programs

Based on screening time, we further divided the data into two periods, before and after 2012. We found that before 2012, the CDR was 1.38‰ (95% CI: 1.08‰–1.71‰), and after 2012, the CDR was 1.26‰ (95% CI: 0.96‰–1.60‰) (Table 3). Similarly, there was little change in the ECDR and the proportion of early-stage cancer over both time periods (Table 4).

### The breast cancer screening effect based on follow-up method in population screening programs

Regarding the follow-up method, the CDR in the no follow-up group was 0.81‰ (95% CI: 0.51‰–1.17‰), which was lower than in the interview follow-up group (1.41‰, 95% CI: 1.15‰–1.69‰) and the second screening group (2.54‰, 95% CI: 1.65‰–3.61‰). The results are summarized in Table 3. Compared to the follow-up screening groups, the ECDR (0.46‰, 95% CI: 0.30‰–0.64‰) and the proportion of early-stage cancer (71.10%, 95% CI: 59.07%–81.95%) in the no follow-up group were also lower (Table 4).

## Discussion

Currently, various breast cancer screening models exist in China, such as population screening, opportunistic screening and physical examinations. However, more and more countries in the European Union are implementing organized screening programs [49]. Organized screening typically has been subjected to rigorous health technology assessment (HTA) to assess its benefits, cost-effectiveness, and potential harmful side effects (although some screening techniques have no adverse reactions) [50]. As a result, China has no real sense of organized breast cancer screening program. CDR and early diagnosis are important indicators to evaluate the quality of cancer screening programs [51]. Our study showed that the CDRs of the two screening models were ≥ 3 times higher than the incidence reported by the Chinese cancer registry [2], and more early-stage breast cancers were detected through screening. Compared with patients with late-stage cancer, those diagnosed with early-stage cancer are more likely to receive curative treatment and have lower treatment costs [52, 53]. Notably, 51.2% of breast cancer patients in the United States were diagnosed with stage I cancer, and more than 84.0% of diagnosed patients had stage 0–II cancer [54]. In high-income Asian countries such as Singapore and Japan, more than 85% of breast cancers were diagnosed at stage II [55, 56]. These data indicate that the proportion of early-stage breast cancer in China is still low. Our findings indicated that the CDR of opportunistic screening was about nine times higher than population screening. However, the proportion of early-stage breast cancer was lower, potentially due to most women participating in opportunistic screening already having noticeable symptoms. Similar results were obtained in other countries [57]. Meanwhile, most opportunistic screenings depend on individual willingness or the extent of available primary healthcare services, thus lacking the guarantee regular screenings. Consequently, population-based organized screening holds greater potential to enhance screening coverage and diminish cancer incidence and mortality rates. Nonetheless, China faces numerous challenges in executing high-quality organized screening, which involve existing infrastructure, resource limitations, and public acceptance of centralized healthcare.

Since the CDR of population screening in meta-analysis is affected by various factors such as strategy of screening, age, population. Therefore, we conducted subgroup analysis to examine the relationship between CDR and these factors. In China, the strategy of population breast cancer screening has undergone a change from using one method alone to using multiple methods in combination. In our study, among the three screening strategies, the CDR for BUS in parallel with MAM was the highest. The proportion of stage 0–II breast cancers was 88.18%, which was consistent with data from other countries [58, 59]. Given China's vast population, diverse economic levels, and disparate resource allocations across regions, implementing a standardized screening strategy poses challenges [60]. BUS, being cost-effective, has gained the main screening method, especially in rural areas of China [61]. At the same time, Chinese women often have smaller breasts with a higher proportion of dense breasts tissue [62]. The latest breast cancer screening guidelines in China recommend BUS combined with MAM for average-risk women with dense breasts or high-risk women [63]. In the future, it's crucial to conduct cost-effectiveness and survival benefit analyses across diverse population screening programs, and establish a systematic national breast cancer screening strategy to standardize the implementation of organized screening programs.

We also found a disparity in preliminary effectiveness of breast cancer screening between urban and rural areas. The CDR in urban areas was about three times higher than that in rural areas, and the proportion of early-stage breast cancer (stage 0–II) could reach 85%. This can be attributed to several factors. Firstly, the incidence of breast cancer in urban areas is higher than that in rural areas. Secondly, women in urban areas possess greater awareness of cancer screening, and have easier access to better medical resources, leading to more diagnoses of early-stage breast cancers [64]. Furthermore, the screening results were also closely related to the geographical location and economic status of the regions. The stage at diagnosis strongly influences the treatment strategies and prognosis of patients with cancer. In China, breast cancer patients consistently have a lower survival rate in rural areas than in urban areas [65]. Enhancing the proportion of early diagnosis might narrow the survival gap among diverse populations. Williams et al. [66] found that women living in non-metropolitan or rural areas were 11% more likely to be diagnosed with late-stage breast cancer than women living in metropolitan or urban areas. The current results suggest that providing free screening services alone cannot compensate for the deficiency in preventive care for low-income and uninsured women [67]. To benefit more women in rural areas, increased clinical services, including follow-ups and medical insurance, are imperative [68, 69].

Chinese women tend to develop breast cancer at an earlier age compared to their Western women. Our findings demonstrated disparities in the starting age of screening, indicating the absence of a standardized criterion for population-based screening in China. The recruitment age of most programs began at 35 or 40 years old, with the detection rate gradually rising with age. However, further survival analysis was lacking, and the benefits of screening at different ages were still uncertain. Studies on the starting age for screening still require a lot of data [70].

The incidence of breast cancer among Chinese women has gradually increased in recent years [2]. Interestingly, we found that the CDR and ECDR of population screening programs did not change significantly over the decade. This trend could potentially stem from publication bias. Moreover, it might be associated with screening management. Although the screening coverage of regions and populations has increased rapidly, there hasn't been a substantial improvement in follow-up methods and service quality. When we further analyzed the follow-up methods, less than 60% of the population screening programs conducted follow-up, and of these, only 43% were published in or after 2012. Without standardization of follow-up management, most high-risk subjects were missed during the program, which substantially reduced the effectiveness of screening [71]. Addressing this issue entails fostering collaboration with cancer registration departments to promptly collect breast cancer incidence, mortality, and survival data. Such data will serve as a critical foundation for conducting comprehensive breast cancer research and health economic evaluations.

Multiple real-world studies have evidenced the positive impact of cancer screening on reducing mortality rates [72–74]. However, a recent meta-analysis on cancer screening suggested that current evidence does not unequivocally establish the life-saving benefits of common cancer screening tests [75]. This prompts us to prudently reassess both the benefits and drawbacks of screening [76]. Notably, not all cancers are suitable for screening. Hence, blindly adopting foreign screening guidelines might not be ideal. Instead, the focus should be on developing screening programs tailored to the specific characteristics of Chinese women. Furthermore, this study corroborates the positive impact of opportunistic screening on elevating breast cancer detection rates. Future endeavors should emphasize heightened publicity and educational campaigns aimed at enhancing women's awareness of breast health and fostering their active participation in screening. Since 2017, our team has carried out a population-based breast cancer screening and intervention technology research program across Liaoning, Shandong, and Shanghai. The program established the first “Program Team-Community-Subjects” network interaction platform to standardize the screening and follow-up process, and applied the latest imaging techniques (digital breast tomography, ultrasonic elastography, and micropore imaging) to compare with conventional techniques (full-field digital mammography and breast ultrasound) in breast cancer screening. This program evaluates the optimal screening strategy for Chinese women and provides a reference for breast cancer screening in China and globally.

### Limitations

There were several limitations to this study. First of all, we found that most of the studies only calculated the CDR without describing TNM staging or early-stage breast cancer detection. Therefore, the studies that could be included were limited, and the description of the CDR may suffer from inclusion bias. Second, the physicians or technicians involved in screening were required to have uniform technical training or qualification, but we did not make subgroup analysis about the facilities used or the professional titles of diagnostic doctors, etc. There may be some bias in the results. Third, the purpose of cancer screening is to find not only early-stage cancer, but also precancerous lesions, especially precancerous lesions that can be treated. We should also analyze the detection of precancerous lesions of breast cancer, but the relevant data of the available studies were limited, so we did not include them.

## Conclusions

In conclusion, there were significant differences in the detection rates of breast cancer and early-stage breast cancer between opportunistic and population screening programs among Chinese women. The results of these population screening studies were influenced by various factors including the screening process, age, population, and follow-up method. Moving forward, China's breast cancer prevention and control efforts should emphasize the advancement of population-based organized screening programs, complemented by opportunistic screening. This strategic approach aims to expand screening coverage and improve screening services.

### Supplementary Information

**Additional file 1: Table S1.** The summary of detailed search keywords. **Table S2.** PRISMA checklist. **Table S3.** The pooled breast cancer detection rates in different subgroups of organized screening programs (China, 2010-2023). **Table S4.** The pooled early-stage (0–II) breast cancer detection rates in different subgroups of organized screening programs (China, 2010-2023). **Table S5.** The pooled proportion of early-stage (0–II) breast cancer in different subgroups of organized screening programs (China, 2010-2023). **Figure S1.** Forest plot of pooled breast cancer detection rate (China, 2010-2023) (A) opportunistic screening; (B) population screening. **Figure S2.** Forest plot of pooled early-stage (0–II) cancer detection rate (China, 2010-2023) (A) opportunistic screening; (B) population screening. **Figure S3.** Forest plot of pooled the proportion of early-stage (0–II) cancer (China, 2010-2023) (A) opportunistic screening; (B) organized screening.

## Data Availability

The original contributions are included in the article. Further inquiries can be directed to the corresponding author.
